# Supplementary material for: Generation of Cytotoxic T Cells and Dysfunctional CD8 T Cells in Severe COVID-19 Patients
Source: Cells. 2022 Oct 25;11(21):3359. doi: 10.3390/cells11213359 (PMC9659290; doi:10.3390/cells11213359)
Supplement: Supplementary file 1 [file cells-11-03359-s001.zip › cells-1910249-supplementary.pdf]

Supplementary Material

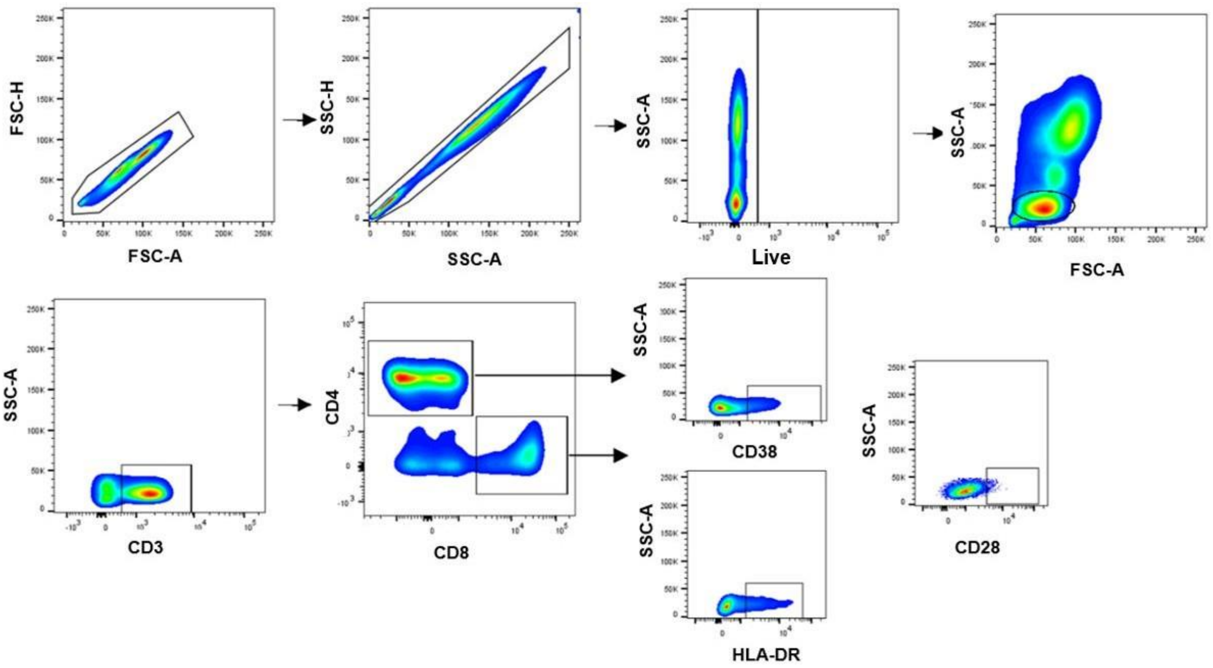

Supplementary Figure S1. Peripheral blood T-lymphocyte analysis strategy. Initially the singlets were selected for FSC and SSC, then the population of live cells. Within the living cells, the lymphocyte population was selected and later the CD3+ population. The CD4+ and CD8+ populations were identified and subsequently evaluated by markers CD38, HLA-DR, and CD28.

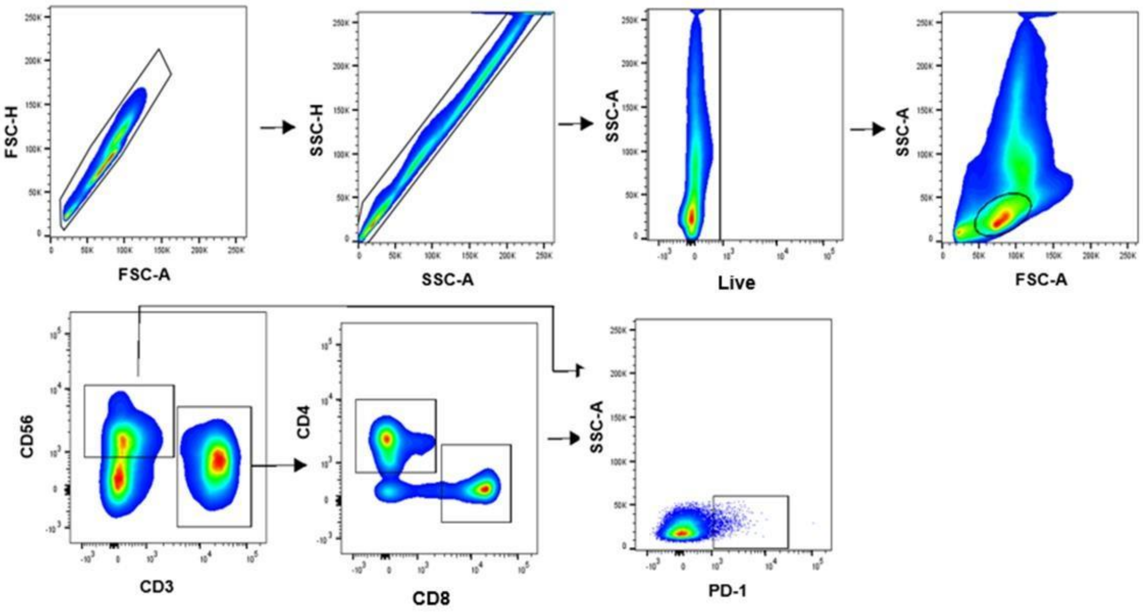

Supplementary Figure S2. Analysis strategy for evaluating PD-1 expression in PBMC. Initially the singlets were selected for FSC and SSC, then the population of live cells. Within the living cells, the lymphocyte population was selected, and later the CD56+ and CD3+ populations were identified. Within the CD3+ population, the CD4+ and CD8+ T-cell gates were selected. In the CD4+ and CD8+ populations, the expression of PD-1 was evaluated by SSC-A.

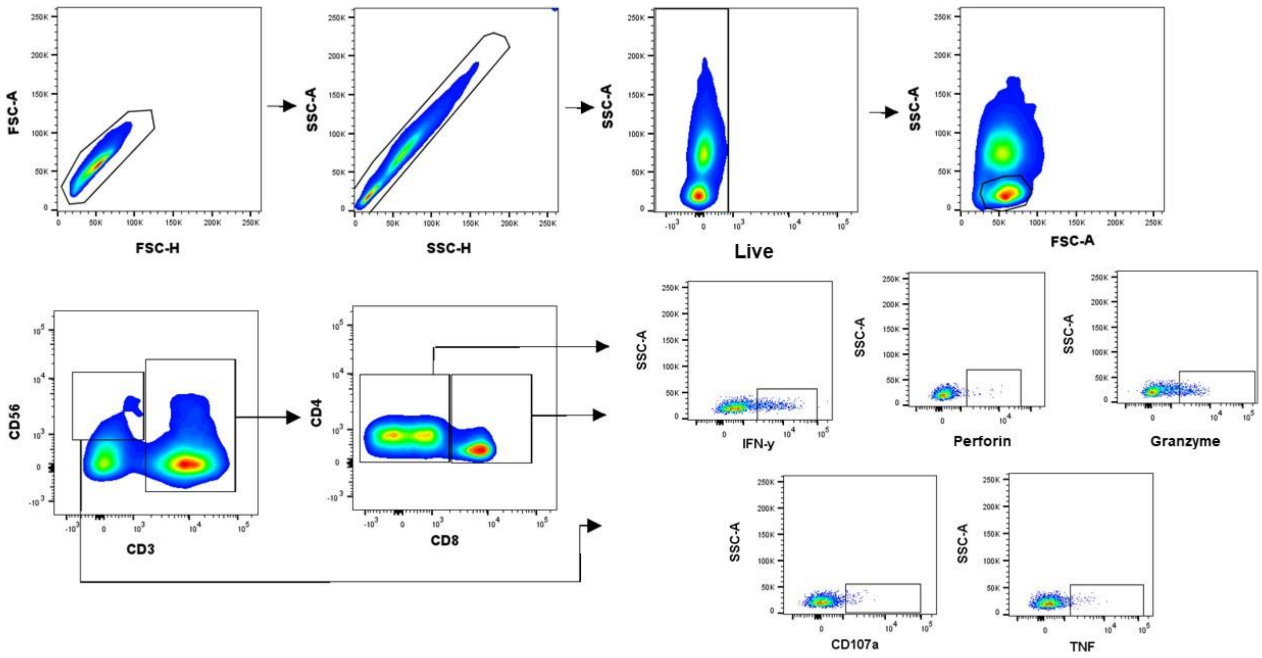

Supplementary Figure S3. Analysis strategy for the functional assessment of CD4+ T-lymphocytes and CD8+ T-lymphocytes. Initially the singlets were selected for FSC and SSC, then the population of live cells. Within the living cells, the lymphocyte population was selected, and later the CD56+ and CD3+ populations were identified. Within the CD3+ population, the CD4+ and CD8 + T-cell gates were selected. In the CD4+ and CD8+ populations, the frequency of IFN-α, Granzyme, Perforin, CD107a, and TNF was evaluated by SSC-A.

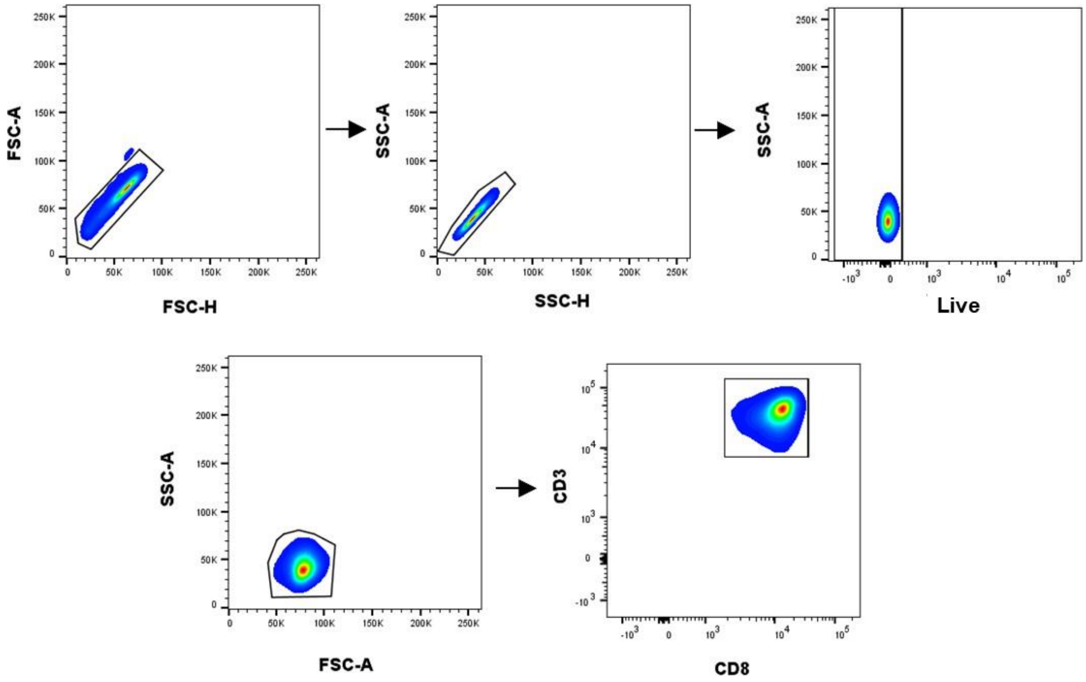

Supplementary Figure S4. Analysis strategy for assessing the purity of CD8+ T-lymphocytes. Initially, the singlets were selected for FSC and SSC, then the population of live cells. Within the living cells, the lymphocyte population was selected and later the TCD8+ CD3+ lymphocyte populations were identified.

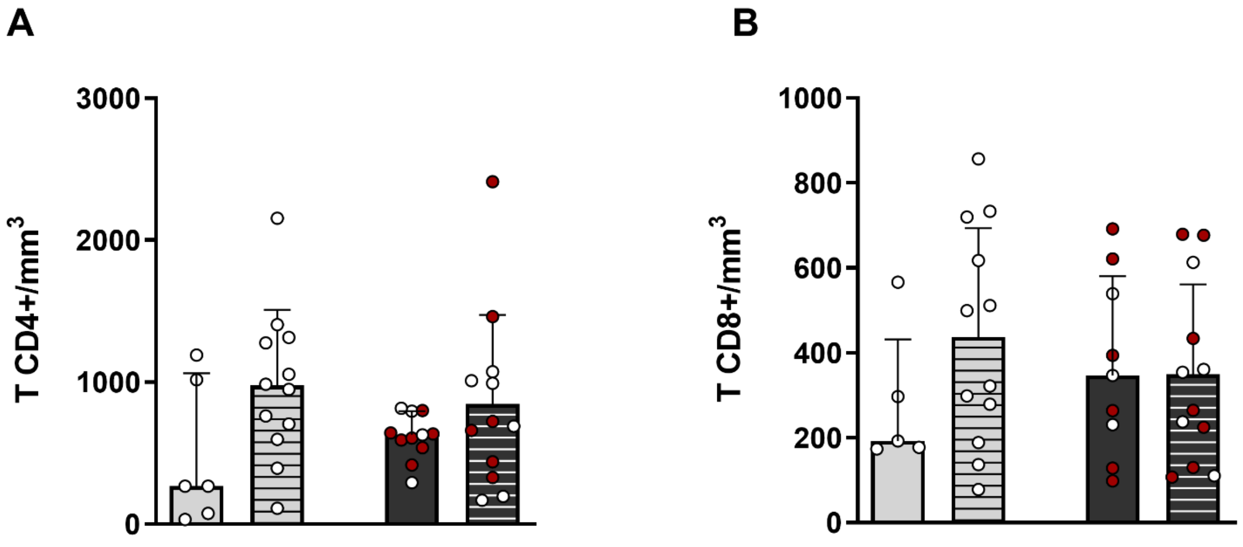

Supplementary Figure S5. Number of CD4+ and CD8+ T lymphocytes in positive PCR periods. The graphs show the number of CD4+ and CD8+ T lymphocytes from SARS-CoV-2 infected patients with moderate or severe/critical disease between 1-7 days positive and 8-20 days PCR positive. The red dots represent patients with the critical infection. The bars represent the median and the interquartile range.

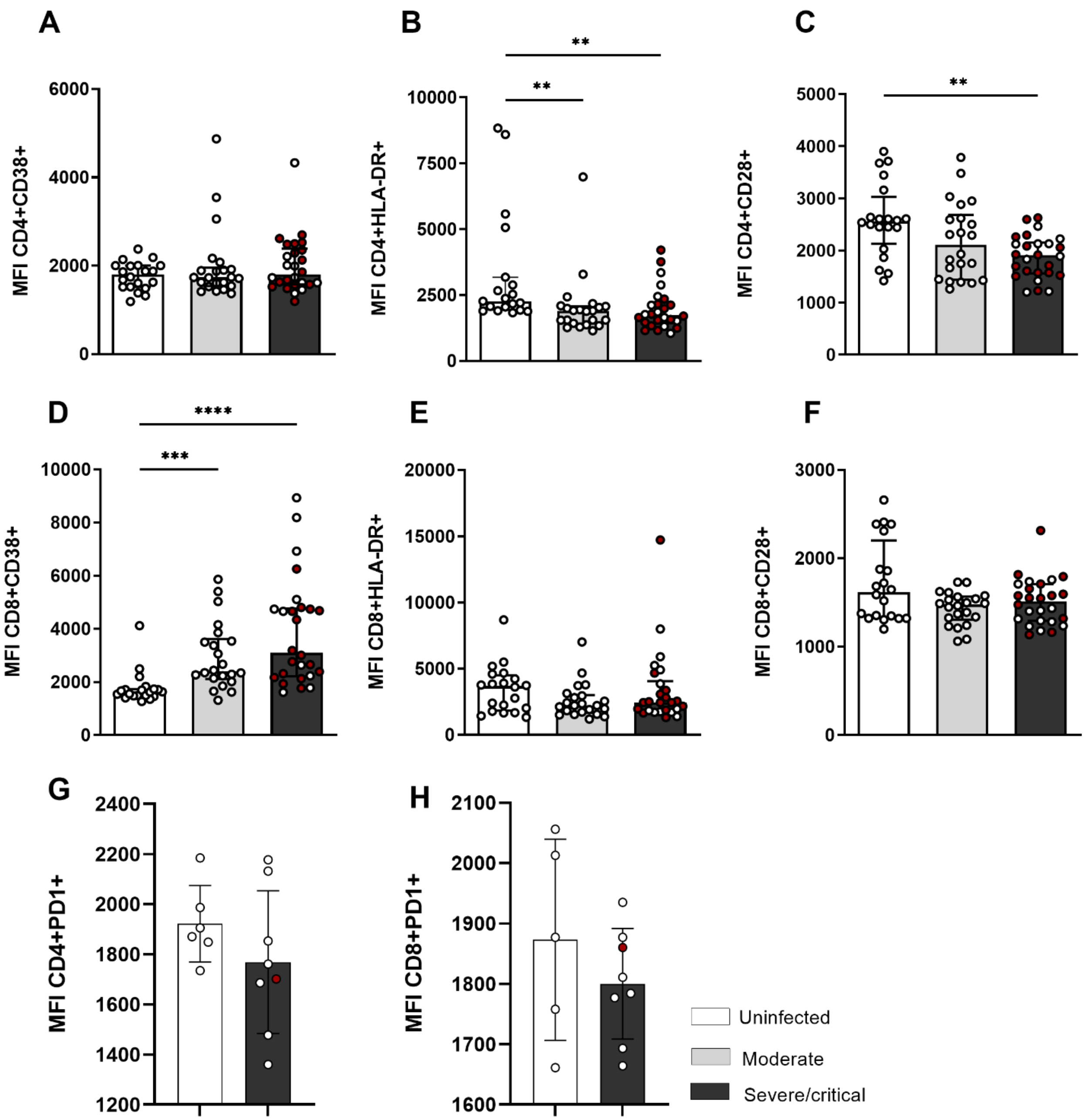

Supplementary Figure S6. Median Fluorescence Intensity (MFI) of activation markers in T- lymphocytes from individuals with COVID-19. The graphs show the MFI of (A) CD38+ in CD4 + T-lymphocytes, (B) HLA-DR+ in CD4+ T-lymphocytes, (C) CD28+ in CD4+ T-lymphocytes, (D) CD38+ in CD8+ T-lymphocytes, (E) HLA-DR+ in CD8+, and (F) CD28+ T-lymphocytes on CD8+ T-lymphocytes in uninfected individuals from patients with moderate and severe/critical disease. PD-1 MFI of PBMCs from (G) CD4+ T-lymphocytes, (H) CD8+ T-lymphocytes from uninfected patients with severe/critical COVID-19. The red dots represent patients with the critical infection. The bars represent the median and interquartile range. ** P < 0.01, *** P < 0.001, and **** P < 0.0001.

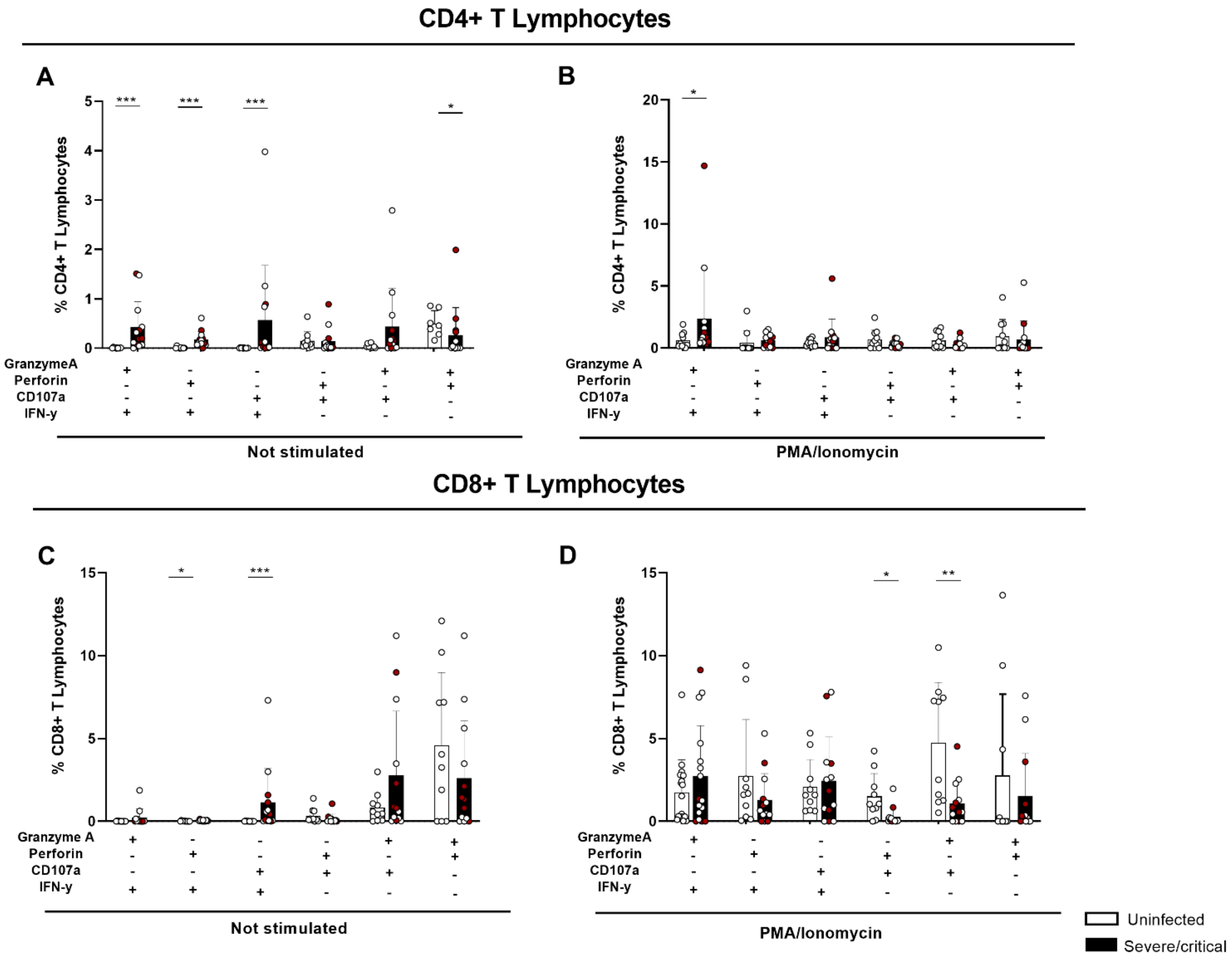

Supplementary Figure S7. Evaluation of the cytotoxic profile of CD4+ and CD8+ T lymphocytes in SARS-CoV-2 infection, considering the double positive. The graphs represent the cytotoxic profile (granzyme A, perforin, CD107a, IFN-γ, TNF) of CD4+ and CD8+ T lymphocytes from PBMCs from control subjects and those affected with severe/critical COVID-19, considering the double positives. (A) Cytotoxic profile of CD4+ T lymphocytes, from basal levels and (B) with stimulation with PMA and Ionomycin, (C) Cytotoxic profile of CD8+ T lymphocytes, from basal levels and (D) with stimulation with PMA and Ionomycin. Stimulated values were subtracted from baseline values. The red dots represent patients with the critical infection. * P<0.0001.

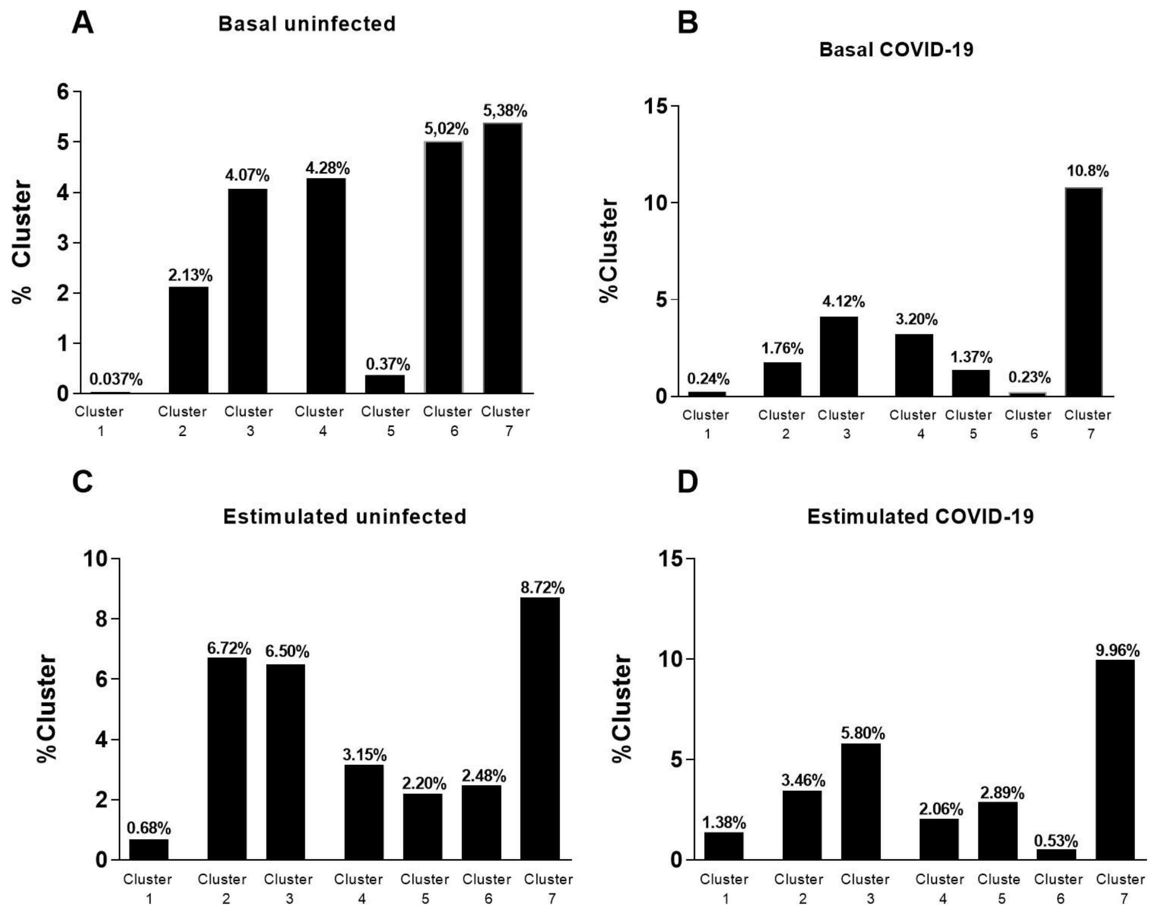

Supplementary Figure S8. Cluster distribution evaluated by tSNE. Percentage of clusters evaluated in the tSNE technique of baseline and stimulated groups of uninfected patients with severe/critical COVID-19.
